# Metacognition impairment in stroke

**DOI:** 10.3389/fneur.2025.1501419

**Published:** 2025-03-11

**Authors:** Wai Kwong Tang, Edward Hui, Thomas Wai Hong Leung

**Affiliations:** ^1^Department of Psychiatry, The Chinese University of Hong Kong, Shatin, Hong Kong SAR, China; ^2^Department of Imaging and Interventional Radiology, The Chinese University of Hong Kong, Shatin, Hong Kong SAR, China; ^3^Department of Medicine and Therapeutics, The Chinese University of Hong Kong, Shatin, Hong Kong SAR, China

**Keywords:** stroke, metacognition, executive function, MRI, prefrontal cortex, anterior insula, amygdala, thalamus

## Abstract

**Introduction:**

Metacognition (MC) impairment is prevalent among stroke survivors but is frequently undiagnosed and untreated. MC impairment hinders stroke survivors’ ability to recognize their deficits, causing them to engage in activities that exceed their capabilities, set unrealistic performance goals and fail to use adaptive compensatory strategies. The present study will evaluate the clinical, neuropsychological and MRI correlates of MC impairment in a cohort of stroke survivors. The secondary objective is to describe the 12-month course of MC impairment.

**Methods and analysis:**

The current study is a prospective cohort study. We will recruit 246 subjects. The project duration is 36 months. Subjects and carers will receive a detailed assessment at a research clinic at three, nine and 15 months after stroke onset (T1/T2/T3). The Chinese version of the Self-Awareness of Deficits Interview (SADI) will be completed by each subject. MC impairment is defined as any SADI subscale score of 2 or more. Potential covariate will be measured as well. Tests of executive functioning will be administered as well. Patients will be examined by MRI within 1 week after the onset of stroke. A stepwise logistic regression will be performed to assess the importance of lesions in the regions of interest. To examine neuropsychological functions in MC impairment, regression analysis of the SADI total and subscale scores will be performed using the significantly correlated neuropsychological functions as predictors. To examine the predictors of MC impairment remission, the demographic, clinical and MRI variables of remitters and non-remitters at T2/T3 will be examined by logistic regression.

**Discussion:**

This project will be the first longitudinal study on MC impairment in stroke survivors. The results will shed light on the association between prefrontal cortex and subcortical lesions and MC impairment risk, symptom severity and outcome.

## Introduction

Metacognition (MC), originally described as the ability to “think about thinking,” includes a broad range of mental processes (1). MC involves the continuous integration of information leads to constructing, defining and refining a complex and evolving representation of the self and the others (1) MC is not a singular concept (2) MC is not intentional but include automatic proceedings (3). MC is not confineable to one person’s mind but its intersubjective in nature (4). MC is not just linked to thoughts about oneself, but are expandable to others (4). MC can be more comprehensively defined as an “umbrella concept” aggregating elements ranging from discrete cognitive processes to more elaborated functions which also include neurocognitive and social cognitive abilities (5).

In the context of stroke, metacognition refers to individuals’ conscious awareness of their cognitive, physical, or emotional functioning and the regulation of these activities through self-monitoring (6). The first major component of MC is self-awareness and the second component is the utilization of self-regulatory and compensatory strategies, including cognitive monitoring and cognitive control (6). Several comparable terms have emerged, such as self-awareness, self-monitoring, self-regulation, subjective cognitive complaints, metamemory or metacognitive awareness (6, 7). Crosson et al. (8) proposed a hierarchical model of self-awareness. The first level, intellectual awareness, is the ability to acknowledge impairment of a body function. Emergent awareness, the next level, is the ability to use this self-knowledge to identify problems with performance of everyday activities. Anticipatory awareness, the highest level of the hierarchy, is the ability to anticipate problems that are likely to occur during activity because of a specific deficit. Toglia and Kirk expanded on this model to include an element of metacognition. Their Dynamic Comprehensive Model of Awareness emphasizes online awareness, an interactive process of performance monitoring and self-regulation during an activity, including the ability to manage environmental and task demands. They also suggested that the components of awareness are less hierarchical and more interdependent (9). Patients with impaired self-awareness engage less in rehabilitation and less often use compensatory strategies (10). Therefore, it is not surprising that impaired self-awareness is associated with low rates of living independently, high rates of family stress, and poor vocational and community outcomes (10). MC impairment can be observed in relation to a wide variety of impairments and phenomena, ranging from physical/motor problems to cognitive deficits (e.g., poor memory) and behavioral disturbances (e.g., apathy) (11). MC impairment is common in patients with cerebral diseases such as dementia (12, 13), traumatic brain injury (11, 14) and stroke (15–17). For instance, in patients with traumatic head injury, 30–67% had MC impairment (11, 14).

MC impairments hinder stroke survivors’ ability to recognize their deficits. MC impairment causes stroke survivors to engage in activities that exceed their capabilities, set unrealistic performance goals and fail to use adaptive compensatory strategies (6). MC impairment contributes to poor therapeutic relationship and rehabilitation outcomes (18, 19), functional capacity (20–22) and recovery (23) in stroke survivors. Similarly, MC impairment is associated with unemployment (24), a poorer quality of life (25), reduced compliance with treatment (26), emotional symptoms and adjustments (27, 28) and behavioral disturbances (11) in patients with head injury. Maladaptive metacognition is associated with anxiety and depression symptoms post-stroke (29). Therefore, MC impairment could be considered as a focus of stroke assessment and rehabilitation (6, 22, 30, 31).

Metacognition impairment appears to be common among stroke survivors. Although small-scale studies reported that 35–46% survivors were affected (15, 18, 32, 33), the prevalence of MC impairment in local stroke survivors remains unknown. The clinical correlates of MC impairment associated with stroke include age (34), a lower educational level, stroke severity, cognitive reserve and impairment (32), verbal memory deficits (31), and anxiety and depressive symptoms (28, 35). Correlates of MC impairment in head injury include younger age (36), severity of injury (34, 36), anxiety and depressive symptoms (25, 37, 38) and executive function (6, 34, 39). Neuroimaging correlates of the severity of MC impairment are unknown; however, the severity of post-stroke depression was shown to correlate with the presence of cerebromicrobleeds (40). Similarly, the remission rate of MC impairment associated with stroke is unknown, although small-scale studies suggested improvements during the first year after stroke (20, 41). Our previous research data yielded a 12-month non-remission rate of 66% for post-stroke depression (PSD), another neuropsychiatric condition, and the clinical correlates of persistent PSD include the baseline severity of depression, severity of stroke and cognitive functioning (42).

The MC impairment is often undiagnosed and thus untreated. Interventions like omega-3 supplementation, viloxazine, and atomoxetine may improve MC in youth with mood disorders or in adults with attention deficit/hyperactivity disorder (43–45). No pharmacological treatment has been found to effectively treat MC impairment in patients with neurological disorders. However, awareness interventions (10, 46, 47), virtual reality games (48, 49) and feedback interventions (50) may be useful treatments for MC impairment. Specific psychotherapies for MC impairment include metacognitive reflection and insight therapy (51), metacognitive grief therapy (52) and metacognitive executive function training (53).

Studies on healthy individuals and lesion studies have suggested that the following areas are important for MC: the orbitofrontal cortex (OFC), dorsolateral prefrontal cortex (DLPC), anterior and posterior cingulate cortex (ACC and PCC), anterior insula and thalamus, particularly the right side (12, 54, 55). MC impairment in dementia is related to OFC and PCC atrophy, hypometabolism and reduced connectivity (12, 54, 56). Lesion studies have suggested that ACC is involved in MC impairment (57). Functional imaging studies indicate that OFC, DLFC, ACC, and PCC is involved with introspection in healthy participants and patients with traumatic brain injury and dementia (12, 54, 56, 58). MC impairment in dementia is related to atrophy of the insula (54, 56). Finally, MC impairment is related to right/bilateral thalamic lesion in stroke (59, 60) and to thalamic atrophy in dementia (54). Our team previously reported an association of thalamic lesions with emotional lability after stroke (61).

Very few structural brain imaging studies have been published on MC impairment in stroke (18, 32, 57, 59, 62). Case reports have linked MC impairment in stroke to infarcts of the right anterior cingulate cortex and right thalamus (57, 59). Cross-sectional studies have associations between MC impairment in stroke and hemispheric lesions (32), lesions in the frontal and temporal lobes, and with lesion size (18, 62). The limitations of these studies include small sample sizes (18, 57, 59), selective sample (62); the lack of a standardized assessment of MC impairment (18, 32) or a detailed examination of infarct locations (18, 32) and no follow-up assessment (18, 32, 57, 59).

The objectives of the proposed study will contribute to the evaluation of the clinical, neuropsychological and MRI correlates and 12-month course of MC impairment in a cohort of stroke survivors. The regions of interest (ROIs) will include the right OFC, DFPC, ACC, PCC, anterior insula and thalamus, and the occipital lobe will be the control region.

### Hypotheses

The first hypothesis is that subjects with MC impairment will harbor more infarcts relative to unimpaired subjects in the ROIs, but not in the control region. The second hypothesis is that there will be a significant positive correlation between the number and volume of infarcts in the ROIs and the severity of MC impairment. The third hypothesis is that subjects with MC impairment will exhibit poorer executive functioning. A positive correlation is predicted between the Self-Awareness of Deficits Interview (SADI) total and subscale scores and executive function in the MC impairment group. The fourth hypothesis is that 66% (42) of subjects with MC impairment at baseline will continue to exhibit MC impairment at 12 months after the first assessment. The fifth hypothesis is that the baseline severity of MC impairment, severity of stroke and level of executive functioning will predict the persistence of MC impairment (42).

## Methods and analysis

### Recruitment of subjects

**Figure 1:**
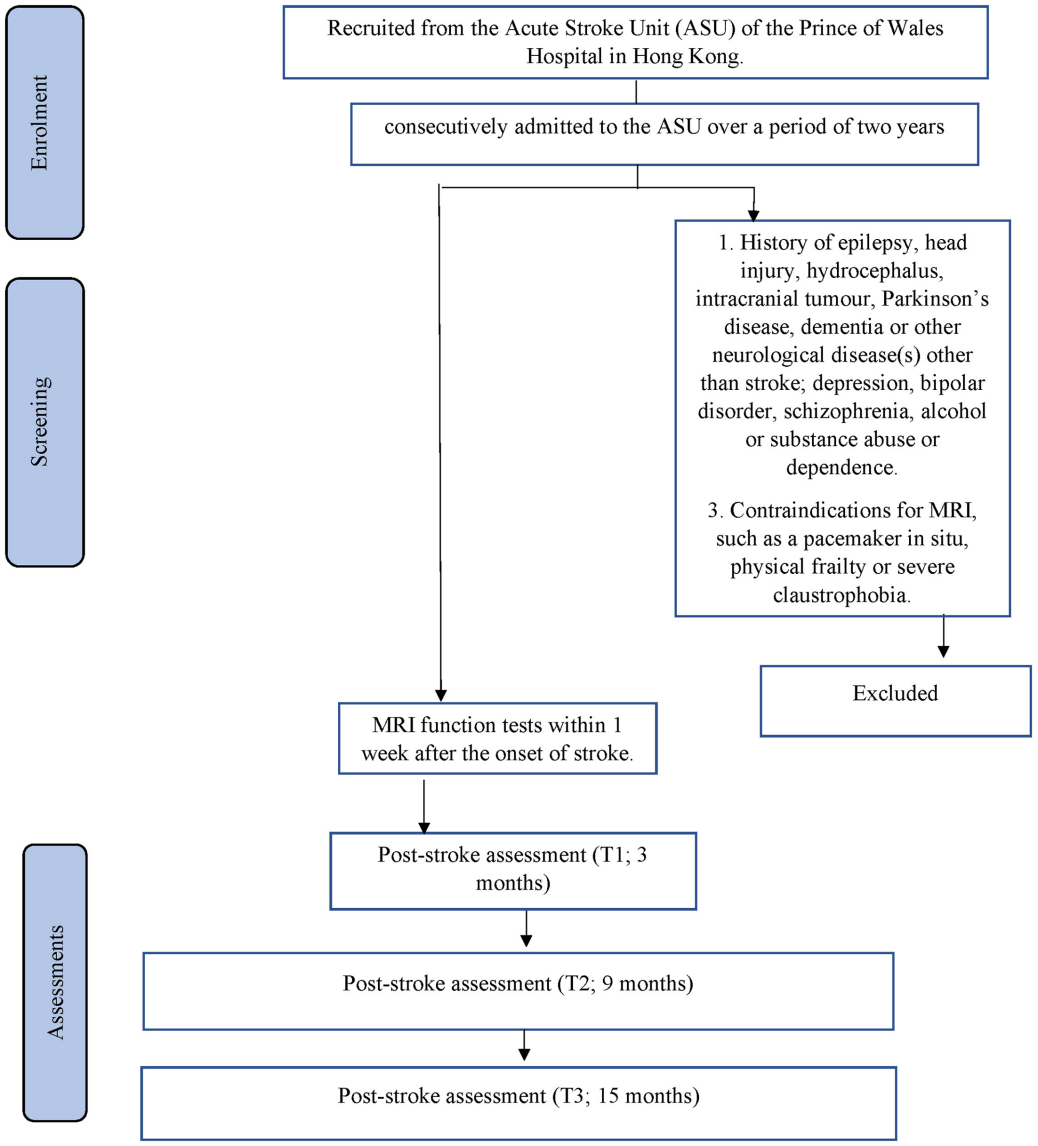
Details of recruitment.

The planned study is a prospective nested case–control study of stroke survivors. Details of recruitment are shown in Figure 1. Patients will be recruited from the Acute Stroke Unit (ASU) of the Prince of Wales Hospital in Hong Kong. The ASU treats approximately 93% of all acute stroke patients admitted to the hospital, with the majority of the remaining 7% sent to the neurosurgery unit. A research assistant will visit the ASU daily to identify all eligible patients and obtain their written consent. All acute stroke patients (*n* = 600) consecutively admitted to the ASU over a 13-month period will be invited to participate. A research assistant will visit the ASU daily to identify eligible patients and to obtain written consent. Approximately 80% of the 600 patients (480) will have ischaemic stroke. MRI examination will be contraindicated in 10% of patients, leaving 432 potential subjects (480 × 90%). According to our previous findings (63), the mortality rate at 3 months post-stroke is around 12%, leaving 380 [432 × (100% − 12%)] potential subjects. Of these, 25% will not meet the inclusion criteria (63). Hence, the number of possible subjects will be around 285 [380 × (100% − 25%)] (63). Assuming a dropout rate of 20%, 228 [285 × (100–20%)] patients will complete the 12-month follow-up assessment. A healthy control group (*n* = 285) matched by age, sex, years of education, socioeconomic factors and ethnicity will be recruited from the community. There is no overlap between the patient samples that will be included in the current manuscript and the other manuscript (MS ID 1451431).

### Eligibility criteria

#### Inclusion and exclusion criteria

The inclusion criteria are:

1. Age >/= 18 years with no upper age limit;
2. Either male or female;
3. Chinese ethnicity;
4. Right handed;
5. Well-documented acute first ischaemic stroke affecting either the right or left hemisphere occurring within a maximum of 7 days prior to admission; and.
6. The ability and willingness to give informed consent.

The exclusion criteria are:

1. Previous history of epilepsy, head injury, hydrocephalus, intracranial tumor, Parkinson’s disease, dementia or other neurological disease(s) other than stroke;
2. History or current diagnosis of depression, bipolar disorder, schizophrenia or alcohol/substance abuse/dependence;
3. Dementia, defined as a Mini-Mental State Examination score below 17 for illiterate individuals, 20 for those with 1–6 years of education and 24 for those with 7 or more years of education (64);
4. Severely impaired language comprehension or expression that precludes a detailed neuropsychological assessment (54);
5. Contraindications for MRI, such as a pacemaker *in situ*, physical frailty or severe claustrophobia;
6. Lack of a caregiver; and
7. Recurrence of stroke prior to the 3-month assessment. For the control group, the exclusion criteria will include a history of stroke.

### Data collection

Details of the data collection schedule are shown in Table 1. Written consent from patients or relatives (by proxy) will be obtained. The number of exclusions and reasons for them will be recorded. The following demographic, psychosocial and medical data of all participants will be collected: age, sex, education, prior history of hypertension, diabetes mellitus and hyperlipidemia and psychiatric disease and date of onset of stroke. The subjects’ clinical data and information on neurological impairment, including dysarthria, measured by the National Institute of Health Stroke Scale (NIHSS) (65), will be extracted from the Stroke Registry. The Stroke Registry is maintained by a full-time, well-trained research nurse. The following strategies will be implemented to reduce attrition: (a) reminder emails and letters will be sent to subjects; (b) five hundred Hong Kong dollars will be offered upon the completion of all follow up assessments.

**Table 1:** Data collection schedule.

Study period	Assessment (T1)	Follow-up (T2)	Follow-up (T3)
Visit	1	2	3
Months after stroke	3	9	15
Review of inclusion/exclusion criteria	X		
Informed consent	X		
Demographics, vascular risk factors, stroke characteristics	X		
Digit Span Test, HKLLT, Go-No-Go test, Wisconsin Card Sorting	X		
SADI. MoCA, BDI, HADSA, FIM, IADL, BI	X	X	X

### Assessment of MC impairment

Three months after the onset of the index stroke (T1), the patients and their carers will undergo the following assessments at a research clinic. The planned timing of the assessment is consistent with other studies of MC impairment in stroke (20, 33).

A trained research assistant (RA) blinded to the subjects’ radiological data will conduct interviews at a research clinic. MC impairment will be assessed based on interviews with the subjects, using the Chinese version of the SADI. The RA will measure each patient’s level of functioning using the Functional Independence Measure (FIM) (66), Instrumental Activities of Daily Living Scale (IADL) (67) and Barthel Index (BI) (68). To assist with scoring, a carer will also complete a checklist regarding the subject’s post-stroke changes, as recommended by Fleming et al. (69).

The SADI is a standardized instrument used to assess the nature of self-identified goals and the ability to set realistic goals. This interviewer-scored, semi-structured interview assesses MC using three subscales, including (i) self-awareness of deficits; (ii) self-awareness of functional implications of deficits and (iii) the ability to set realistic goals (69). Each component is scored by a therapist on a four-point Likert scale ranging from zero (no awareness) to three (complete awareness). An example question is “Are you any different now, compared to what you were like before the stroke?” For each subscale, lower scores (0–1) reflect moderate to high MC. Conversely, higher scores (2–3) reflect a low MC (i.e., tendency to deny or minimize the extent of difficulties experienced) (46). The three sub-scale scores can be summed to yield a SADI total score that ranges from 0 to 9, with higher scores indicating lower metacognitive awareness. The duration of administration is 20–30 min (70). MC impairment is defined as any SADI subscale score of 2 or more (46, 71). The interrater reliability coefficients were found to be 0.78, 0.57 and 0.78 for the three subscales and 0.82 for the total SADI score (69). The construct validity of the SADI is supported by significant correlations of this instrument with other standardized measures of self-awareness (27, 72). The SADI also has satisfactory inter-rater (0.82) and test–retest reliability (0.85–0.86) (73) and responsiveness (70). The SADI has previously been used to assess MC impairment in stroke patients (10, 59), and is currently the only standardized self-awareness measure that investigates a client’s ability to set realistic goals (69). The Dysexecutive Questionnaire score (74) was not found to correlate with the SADI score (72). Other scales used to measure MC impairment, such as the Patient Competency Rating Scale (75) and Self-Regulation and Skills Interview (46), have not been translated into and validated in Chinese; whereas the Awareness Questionnaire (76, 77) has not been applied in stroke patients.

A trained RA, blind to the subjects’ radiological data, will administer the following neuropsychological tests (Table 1).

### Assessment of basic cognitive functioning

1. The Chinese version of the Montreal Cognitive Assessment (MoCA) (78) measures global cognitive functions. MoCA is a 30-point scale that covers multiple cognitive domains including spatiotemporal orientation, sustained attention, visuospatial function, executive function, verbal memory, language, naming, and abstract thinking. MoCA showed adequate sensitivity and specificity for the detection of post-stroke cognitive impairment (78).

2. The Digit Span Test (79) assesses attention and working memory. This test requires subjects to repeat series of digits of increasing length. Digit span forward is a good measure of simple attention, and most healthy individuals perform within the seven plus/minus two span of apprehension range. The digit backward sub-score, which ranges from 0 to 14, will be used as the index of working memory. A higher score reflects superior performance. Digit Span Test has been commonly applied in stroke studies (80).

3. The Hong Kong List Learning Test is used to assess memory (81). This test, which is based on the California Verbal Learning Test, presents 16 words in 3 learning trials, followed by a 10-min delayed recall and 30-min delayed recall and recognition test. It has been validated in both normal and clinical samples (81). The Hong Kong List Learning Test has been applied in local patients with stroke and neurocognitive disorders (82, 83).

### Assessment of executive functioning

1. The Go–NoGo test (84) assesses response selection/inhibition. A motor response (pressing a button as quickly as possible) is either initiated (Go) or inhibited (NoGo), depending whether a stimulus comprising a green square (Go) or red square (NoGo) appears on a computer screen. Errors of omission (misses) and commission (false alarms) and the reaction time needed to correct trials during the experimental condition are recorded. The Go–NoGo test had been applied in patients with stroke (85, 86).

2. The Wisconsin Card Sorting Test (87) is composed of 4 stimulus cards and 64 response cards. The response cards differ in three dimensions: color (red, green, yellow, or blue), pattern (triangle, star, cross, or circle) and number (one, two, three, or four). The participant is asked to work out a sorting principle for matching each response card to the four stimulus cards (one red triangle, two green stars, three yellow crosses or four blue circles) according to the feedback given by the examiner (correct or incorrect). Once the participant has made 10 consecutive correct matches to the sorting principle, the sorting principle is changed without warning and the participant is required to work out a new sorting principle. The test is terminated when the participant has: (1) successfully maintained 6 correct sorting principles (color, pattern, number, color, pattern, number) or (2) made 128 attempts. To evaluate the participant’s abilities to use abstract reasoning and shift cognitive strategies, each response is recorded as either correct, a perseverative response, a perseverative error or a non-perseverative error for subsequent scoring. The number of completed categories, total number of attempts and number of perseverative errors are selected as the index scores. The Wisconsin Card Sorting Test has been validated and applied in patients with stroke (88, 89).

### Other assessments

A trained RA will measure the level of depressive and anxiety symptoms using the Beck Depression Inventory (BDI) (90) and the anxiety subscale of the Hospital Anxiety Depression Scale (HASDA) (91), respectively. BDI is a 21-item, self-report rating inventory that measures characteristic attitudes and symptoms of depression. The BDI takes approximately 10 min to complete. HADSA is 7-item scale. Each item scores on a 4-point Likert scale (e.g., as much as I always do [0]; not quite so much; definitely not so much; and not at all), giving maximum subscale scores of 21. The questionnaire assesses symptoms over the preceding week.

Follow-up assessments of MC impairment will be conducted on all stroke patients at 9 months (T2) and 15 months (T3) post-stroke, or 6 and 12 months after the first assessment. SADI, FIM, BI, MoCA, BDI and HADSA will be repeated during the follow-up assessment.

### Magnetic resonance imaging (MRI) examination and analysis

Patients will be examined by MRI within 1 week after the onset of stroke. All scans will be performed using a 3 T scanner (Philips Achieva 3.0T, X Series, Quasar Dual MRI System). Standardized sequences includes diffusion weighted imaging (DWI), 3D T1-weighted, T2-weighted, fluid attenuated inversion recovery (FLAIR) and susceptibility-weighted imaging (SWI). An experienced neuroradiologist blind to the subjects’ MC impairment status will assess the MRI images. Acute infarct will be defined as a hyperintensive lesion on DWI with corresponding hypointensity on the ADC map. White matter hyperintensities (WMH) will be defined as hyperintensities 5 mm that are ill defined on FLAIR images, but are isointense with normal brain parenchyma on T1 weighted images. Lesions equivalent to the signal characteristics of cerebrospinal fluid on T1-weighted images and measuring more than 3 mm in diameter, and also wedge-shaped cortico-subcortical lesions, will be regarded as old/lacunar infarcts. Microbleeds will be defined as dot-like hypointensities on SWI. The total number of microbleeds will be determined. All raw data will be transferred to the PALS system (Carestream Solutions).

### MRI pre-processing

This will include non-uniformity correction (92), spatial standardization and brain extraction (excluding the skull). To ensure the brain structure volumes are comparable among subjects, the MRI data of each subject will be transformed from the original space to a common stereotactic space using multi-scale affine registration (93). Brain regions will be automatically segmented from the head MRI data using the brain extraction tool (94).

### Brain segmentation

Brain tissue will be classified into grey matter, white matter and cerebrospinal fluid (95). Whole-brain segmentation will be achieved using an atlas-based approach (96), which automatically adjusts the existing atlas intensity model to newly inputted data. The ROI and other brain regions will be segmented and their volumes quantified using the Talairach brain atlas (97) and Daemon registration.

### Infarct segmentation and quantification

Infarcts will be delineated semi-automatically as high-intensity regions on DWI images and WMH as high-density regions on FLAIR images (and isointense on T1 weighted images) using ITK-SNAP software. The segmented infarct and WMH regions will be combined with the ROI and other brain-region masks generated in the previous step. The infarct and WMH pixels that fall within the ROI and other brain regions can then be calculated.

### Sample size estimation

Two hundred and eighty-five patients will be recruited. A previous report of MC impairment in stroke found that 47% of patients with MC impairment presented with frontal infarcts, compared to only 10% of patients without MC impairment (18). The corresponding effect size of frontal infarct would then be 0.415. A sample size of 285 will have a power of 99% for the identification of frontal infarcts as a predictor of MC impairment in stroke, based on a chi square test with one degree of freedom (98).

Using 46% (36) as an estimate of the frequency of MC impairment, 131 (285 × 46%) cases will be identified. A sample size of 131 subjects with MC impairment would provide a power of 80% (99) for detecting any correlate of the SADI total score with a correlation value of 0.14. This magnitude of correlation is considerably lower than the reported associations between the severity of MC impairment and severity of injury (0.40) (100), emotions (0.21) (25), verbal memory deficits (0.81) (31) number of lesions (0.30) (58) and executive functions measured with Wisconsin Card Sorting Test perseverative responses and errors (0.28–0.31) (39) and Go–NoGo test omission and commission errors (0.19–0.28) (101).

Assuming a drop-out rate of 20%, 105 (131 × 80%) patients with MC impairment will attend the follow-ups. Although the remission rate of MC impairment is unknown, previous studies have reported remission rates of 35–50% in PSD (42). Therefore, this sample will provide a power of 80% for identifying any predictor with an odds ratio of 2.0. This odds ratio is lower than the figure of 5.3 reported in our previous work on cerebral microbleeds and PSD outcomes (42), assuming that the R2 of the other variables (stroke severity, depressive symptoms, cognitive function) is 0.21 (42) in the multivariate logistic regression (98).

### Statistical analysis

Missing data will be handled by the last observation carried forward method. All of the variables will be tested for normality using Kolmogorov–Smirnov tests with a significance threshold of *p* < 0.05. We will first compare the SADI and other neuropsychological test scores between stroke subjects and normal controls. To examine the correlates of MC impairment, the demographic, clinical and MRI variables (age, education, prior history of hypertension, diabetes mellitus, hyperlipidemia and psychiatric disease, NIHSS, HADSA BDI, MoCA, ROI infarcts, microbleeds and WMH volumes) will be compared between patients with and without MC impairment at T1. The *χ*^2^ test, Student’s *t*-test or Mann–Whitney *U* test will be applied as appropriate. A stepwise logistic regression will be performed to assess the importance of lesions in the ROIs, together with other significant variables in the above univariate analyses, effect sizes and confidence intervals will be computed. For the patients with MC impairment at T1, the SADI total and subscale scores for groups with and without ROI infarcts will be compared using a covariance analysis.

To examine neuropsychological functioning in MC impairment, the performance of the groups with MC impairment, without MC impairment and the normal controls on executive and basic cognitive function tasks will be compared using analysis of variance. The correlation between the SADI total and subscale scores and the performance of the group with MC impairment on the above neuropsychological tests will be computed using Pearson’s or Spearman’s correlation coefficients, as appropriate. Finally, a regression analysis of the SADI total and subscale scores will be performed using significantly correlated neuropsychological functions as predictors. To examine the interplay between lesion location and severity, executive function and depressive symptoms, interaction terms will be created and analyzed.

To examine the predictors of remission of MC impairment, the demographic, clinical and MRI variables of remitters and non-remitters at T2/T3 will be examined using logistic regression. We will also test a series of generalized estimating equation models to evaluate the associations of clinical and brain MRI characteristics with the risk of MC impairment across all follow-up assessments (T1/T2/T3). First, we will run a univariate model to fit a logistic regression. Next, we will examine the associations between the demographic variables, concurrent medical conditions and risk of MC impairment. The second model will include the baseline SADI scores, NIHSS scores and cognitive and executive function scores. Brain MRI characteristics will be entered in the final model, effect sizes and confidence intervals will be computed. The level of significance will be set at 0.05.

Structural equation modeling will be applied to help elucidate causal pathways to remission of MC impairment. *A priori* model is constructed with explicit root causes (demographic, clinical and MRI variables) and mediators (MC impairment severity, depressive and anxiety symptoms, functional impairments) leading to remission. Following structural equation modeling, a posteriori model is selected based on data fit and clinical plausibility.

## Discussion

We try to achieve a homologous patient population by narrowing the age, ethnicity, handedness and duration of MC impairment. Patients with other causes of MC impairment, such as psychiatric or neurological disorders are excluded. This project will be the first longitudinal study to examine the role of OFC, DFPC, ACC, PCC, anterior insula, amygdala and thalamus in a large sample of consecutively admitted stroke survivors with MC impairment. Compared to previous neuroimaging, this study has large sample size, standardized assessment of MC impairment, detailed examination of infarct locations and no follow-up assessment. The results will shed light on the association between the above brain regions and MC impairment risk. They are thus likely to be applicable to the large population of neurological patients at risk of MC impairment.

The findings should also stimulate and guide further research in this field. For example, the information on neuroimaging correlates will guide the design of functional imaging studies of brain network involved in MC impairment. Whereas knowledge on executive function impairment may facilitate the design of potential non-pharmacological treatment. Finally, information on the rate and predictors of remission will provide useful information on clinical trials.

The findings regarding the relationship between MC impairment and location of infarcts and executive function may have important clinical implications. In fact, studies have shown that the lack of awareness is related to more difficulties with treatment adherence, poorer rehabilitation outcomes and greater emotional symptoms, behavioral disturbances and unemployment, caregiver burden, all of which increase the financial impact on society (11, 19, 24, 26, 27). Interventions that decrease MC impairment may eventually bring important clinical benefits. For instance, the information on the location of infarcts may prompt development of stimulation paradigms of specific brain regions using repetitive transcranial magnetic stimulation or transcranial direct current stimulation. These types interventions have been tested in patients with depression after stroke (102, 103). Alternatively, data on executive function deficits may suggest the possibility of interventions on improving executive functions. This type of cognitive rehabilitation has already been tested in patients with depression after stroke (104).

This study has some limitations. Patients of non-Chinese ethnicity. Who cannot give consent because of dementia or aphasia-associated left-side infarcts, were excluded. This selection bias may limit the generalizability of the findings. Furthermore, unmeasured confounders may affect the results. Finally, none of the patients will receive treatment for their MC impairment.

The current manuscript focuses solely on MC impairment in stroke, while a separate study published in *Front. Psychol. – Neuropsychology* (105) will examine loss of empathy in stroke only. MC impairment and loss of empathy are different domains of neuropsychiatric deficits. The patient cohorts that will be used in these two manuscripts will be entirely different.

## Conclusion

The study protocol will provide a comprehensive overview of the predictors, latent factors, and outcomes of MC impairment in stroke patients. It will also inform clinical practice and advance the rehabilitation of stroke.
